# *Alaria alata* Infection in European Mink

**DOI:** 10.3201/eid1909.130081

**Published:** 2013-09

**Authors:** Flaviu Tăbăran, Attila David Sándor, Mihai Marinov, Cornel Cătoi, Andrei Daniel Mihalca

**Affiliations:** University of Agricultural Sciences and Veterinary Medicine Cluj-Napoca, Cluj-Napoca, Romania (F. Tăbăran, A.D. Sándor, C. Cătoi, A.D. Mihalca);; Danube Delta National Institute for Research and Development, Tulcea, Romania (M. Marinov)

**Keywords:** *Alaria alata*, mesocercariae, European mink, *Mustela lutreola*, larva migrans, parasites

**To the Editor**. Alariosis is a reemerging zoonotic disease caused by infection with larval stages of trematodes of the genus *Alaria*. The trematodes are found in wildlife that inhabit wetlands, and these animals may serve as possible reservoirs for these organisms that cause human infection (*1*). The main sources for human infection are suids and frogs (*1*). In humans, the clinical features of alariosis caused by infections with the North American species of *Alaria* vary from mild and asymptomatic to moderate with respiratory or cutaneous signs (*2*) or neuroretinitis (*3*), to severe-to-lethal anaphylactic shock caused by larva migrans (*4*,*5*). The genus *Alaria* has 7 species; only *A. alata* is found naturally in Europe (*6*), a species which has not thus far been shown to be responsible for human infections.

*A. alata* infection is common in its typical definitive host (red fox, *Vulpes vulpes*) and in certain paratenic hosts (wild boar, *Sus scrofa*) (*1*). However, the role of other paratenic hosts is poorly known. Among these, mustelids are reported to harbor mesocercariae of *A. alata* trematodes (*7*). The pathogenic effect of *A. alata* infection has been poorly studied, because most lesions described were in humans infected with other species of *Alaria*. Except for 2 experimental studies that described gross lesions produced by *A. alata* trematodes (*6*,*8*), to our knowledge, no data have been published concerning lesions produced by natural infection in nonhuman hosts. Our report provides a detailed description of the lesions, shown by microscopy, which suggests the pathogenic mechanisms.

One adult female European mink (*Mustela lutreola*) was found dead during standard surveillance operations in which box traps were used; this trapping was part of biodiversity and ecology studies in the central part of the Danube delta in Romania (45°08′N, 29°19′E) in March 2010. The corpse was deep-frozen and analyzed after 3 months in the laboratory. During necropsy, multiple, well-defined, whitish nodules were observed in most muscular and subcutaneous tissues (Figure, panel A), with no evident preferential localization. We collected samples from these tissues for artificial digestion (*9*,*10*) and histologic examination, using the routine paraffin-embedding protocol and the following staining methods: hematoxylin-eosin, Masson trichrome, and Gordon and Sweet.

Artificial digestion released parasites (6 larvae/5 gm tissue) with typical larval trematode structures (Figure, panel B). By microscopy, we observed that morphologic features of these larvae were consistent with *A. alata* mesocercariae (*6*). Histopathologic examination confirmed the presence of parasitic forms in muscle sections (Figure, panel C). The mesocercariae were located in the connective fibrous tissue of the perimysium or between the muscle fibers. The typical structure of muscle fibers was altered around the larvae, with inflammatory cell reactions, represented mainly by lymphocytes, macrophages, and plasma cells (Figure, panel D). In other areas, the inflammatory reaction around the parasite was minimal or absent (Figure, panel E). In certain histologic sections, the damaged muscular tissue was replaced by granulation tissue in various stages of development (Figure, panel F). The maturity of the granulation tissue differed substantially, depending on the muscular areas examined. Some lesions were found in adult connective tissue, formed by mature collagen scar fibers (type I collagen) and few inflammatory cells, whereas other lesions had reticulin fibers (type III collagen) with numerous inflammatory cells. The lesions of the subcutaneous connective tissue consisted of an inflammatory reaction (panniculitis). The inflammation was characterized by a low number of mononuclear leukocytes and fibrinous exudate and fibroplasia.

The polyphasic nature of muscle and subcutaneous lesions produced by *A. alata* infection in its paratenic host appears to be caused by mesocercarial migration. This view is sustained by the presence of mononuclear cells that it infiltrates and by the appearance of the granulomatous tissue in various stages of maturation, which leads to muscle and subcutaneous fibroplasia. The reparatory nature of the lesions suggests that the inflammation is probably the result of direct tissue damage rather than an immune reaction targeted toward the parasitic antigens. This assumption could explain the local absence of inflammatory reaction around the parasites. The lack of inflammation was previously observed also with *A. americana* infection of humans (*4*). The structure of all mesocercariae observed by microscopy suggested that they were alive and active before the mink carcass was frozen. Because no mesocercariae were surrounded by adult connective tissue or by granulomatous inflammation, together with the multiple presences of migratory routes, the continuous mobility of the parasites through the host’s tissues was strongly suggested.

Although data on the pathologic changes caused by *Alaria* spp. in general, and *A. alata* parasites in particular, are scarce, the migration pattern and the lesions seem to be dependent on the particular parasite and host species. The reparatory nature of the lesions suggests that the inflammation is the result of direct tissue damage rather than an immune reaction targeted toward the parasitic antigens.

**Figure:**
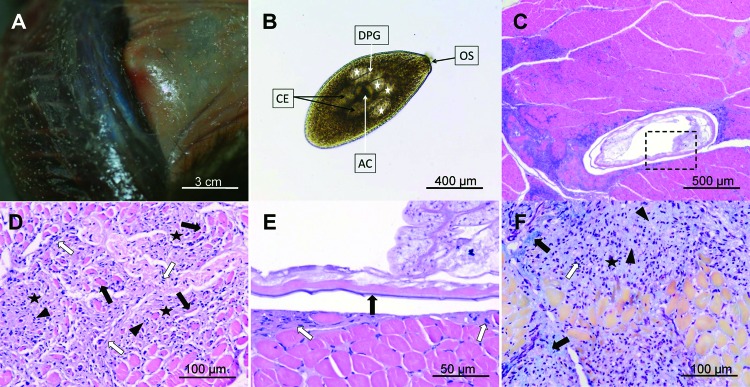
Lesions produced by mesocercariae of Alaria alata in European mink. A) Mesocercariae in the muscle and subcutaneous tissue produce whitish, round or slightly oval, well-defined nodules. B) Free mesocercarium after artificial digestion, showing the characteristics of A. alata mesocercarium: piriform body with anterior oral sucker (OS), acetabulum (AC) positioned in the center of the parasite, 2 pairs of large, finely granulated penetration glands (white stars), limiting the anterior part of the acetabulum, linear ducts of penetration glands (DPG) converging to the oral opening of the oral sucker and large double ceca placed posterior to the acetabulum. C) Histologic section showing an encysted mesocercarium in the muscle (parasite is surrounded by a capsule and pericystic inflammation, which extends to the surrounding muscular tissue). D) Mononuclear leucocytes (arrowheads) scattered between the fibroblastic proliferations (white arrows) and collagen deposits (black stars). Muscle fibers are atrophic due to compression (black arrows). E) Microscopic detail of the inset from panel C. Some mesocercariae (indicated by black arrow) are enclosed in a thin, pale staining capsule (white arrows). Note the lack of leukocyte response. F) Migration route of the parasite (route with center marked by the black star), followed by invasion of nonnecrotic muscle fibers by mononucleate inflammatory cells (white arrow) located mainly in the center of the migration tract and fibrous connective tissue with collagen fibers densely packed at the periphery (bright green, marked by black arrows) and more loosely in the center (pale green material, marked by arrowheads). Hematoxylin–eosin stain (panels C, D, E); Masson trichrome (panel F); original magnifications x40 (panels B and C), x200 (panels D and F), and x400 (panel E).
